# Durability and Moisture Dynamics of Douglas-Fir Wood From Slovenia

**DOI:** 10.3389/fpls.2022.860734

**Published:** 2022-03-29

**Authors:** Miha Humar, Viljem Vek, Primož Oven, Boštjan Lesar, Davor Kržišnik, Eli Keržič, Miha Hočevar, Robert Brus

**Affiliations:** Biotechnical Faculty, University of Ljubljana, Ljubljana, Slovenia

**Keywords:** wood, natural durability, wood decay, water exclusion efficacy, extractives

## Abstract

Wood in outdoor applications is subject to various decomposition factors. Wood degradation can be prevented by construction details, biocide protection of wood, wood modification or selection of naturally durable species. Unfortunately, most species in Europe do not have naturally durable wood. Imported tree species represent a new pool from which we can draw wood species with better natural durability and better resilience towards climate change. The performance of wood when used outdoors depends on the biologically active compounds (extractives) and the water exclusion efficacy. Considering decay, presence of biologically active compounds and water exclusion efficacy, we can estimate the density, modulus of elasticity, extractive content and resistance dose, which reflects the material properties of wood. Recently, the most commonly used model for this purpose is Meyer-Veltrup. Literature data indicate that the durability of the wood from native and new sites is not always comparable, so it is necessary to determine the resistance of non-native wood species from new sites. This paper presents original data on the wood’s overall durability from American Douglas fir (*Pseudotsuga menziesii*) grown in Slovenia. Experimental data show that the mature heartwood of Douglas fir is more durable than the wood of European larch (*Larix decidua*). Durability can be attributed to good water exclusion efficacy and inherent durability. Inherent durability is primarily the result of the high content of extractives. Based on the results, it can be concluded that American Douglas fir grown in Central Europe has a high potential for outdoor use.

## Introduction

The climate is changing. Measurements show a trend towards rising air temperatures. Finally, the last ten years are among the warmest years in terms of weather observation in Slovenia and other central European countries. Moreover, we are experiencing increasingly intense extreme weather events (hail, storms and others; Oblak et al., 2021). We can likely expect even stronger and more pronounced climate change in the future (Bevacqua et al., 2021).

Climate change is already affecting the forest species composition and wood quality (Gričar et al., 2015). Increasingly higher temperatures and a changing precipitation regime are expected to affect tree species’ distribution in these forests significantly. Assuming limited migration, most European species would significantly decline suitable habitat areas. Most biome shifts focus on ecological issues, as changes in dominant tree species will also lead to changes in entire ecosystems and dependent organisms (Dyderski et al., 2018). Generally accepted models suggest that depending on the climate scenario, only the Mediterranean type of low-value oak forest will thrive on 21–60% (average 34%) of European forest area by 2,100. This will result in lower yields for forest owners and a shortage of raw material for the timber industry (Hanewinkel et al., 2013). In addition, it must be considered that CO_2_ sequestration in such modified forests will also be reduced (Birdsey and Pan, 2015). Models showed that *Abies alba*, *Fagus sylvatica*, *Fraxinus excelsior*, *Quercus robur*, *Quercus petraea* and the non-native species *Pseudotsuga menziesii*, *Quercus rubra* and *Robinia pseudoacacia* could be considered climate winners (Dyderski et al., 2018).

Based on the data presented, one viable solution to improving the economic perspective of forest owners and related wood processing industries is the controlled introduction of non-native tree species such as Douglas fir (Eberhard et al., 2021) in Central Europe. The future use of these tree species must also be of long-term interest to the forest owner. The economic impact is primarily based on the relevant properties and the associated price of the forest assortments on the market. Most of the Slovenian wood processing industry is based on softwoods. Softwoods are of crucial importance for timber construction (roofs, skeletal structures, glue-laminated beams and cross-laminated timber) and the composite industry (particleboard and shuttering panels; Ansell, 2015). Today, the wood used for these purposes in Slovenia are mainly Norway spruce (*Picea abies*) and silver fir (*Abies alba*). However, their share in Slovenian forests decreases (Klopčič et al., 2017). Therefore, it is crucial to identify the relevant properties of a suitable wood species that could partially replace Norway spruce in the applications mentioned above. Douglas fir (*Pseudotsuga menziesii*) was defined as a potential candidate among different wood species. Douglas fir is already present in Europe and covers about 830,000 ha of forest area in Europe alone (Pötzelsberger et al., 2020; Eberhard et al., 2021). These tree species offer high productivity rates and thus ensure sustainable income for forest owners (Eilmann and Rigling, 2012).

The first documented introduction of a considerable number of non-native tree species on the territory of present-day Slovenia occurred in the late 18th and early 19th centuries. Initially, the species were planted in specialised plant collections, and the frequency of their planting was low. At the end of the 19th century, the planting frequency of some species increased, and several species were also introduced into forests (Brus and Gajšek, 2014). Most Douglas-fir plantations in Slovenia were established in the periods 1880–1940 and 1960–1990 in various parts of the country (Veselič et al., 2016). Douglas fir is currently the third most common non-native tree species, accounting for 0.05% (171,000 m^3^) of the total growing stock in Slovenian forests. It has clearly shown good adaptability under challenging conditions, e.g., resistance to ice storms, drought and bark beetle infestation in areas where Norway spruce was severely affected, as well as good growth performance. For this reason, Douglas fir has currently considered the most promising non-native tree species in Slovenian forests (Smolnikar et al., 2021).

The European standard EN 350 (CEN, 2016) clearly distinguishes the durability of Douglas fir from North America from Douglas fir growing in Europe. The durability of Douglas fir from North America is 3 (moderately durable), while the durability of Douglas fir grown in Europe has a higher variation of 3–4 (slightly to moderately durable). Douglas-fir wood is generally characterised as the species with one of the best ratios between growth rates and quality (Perić et al., 2011). The wood properties are determined by numerous factors: site conditions, provenance, forest management and location in the tree (Pollet et al., 2017; Balanzategui et al., 2021; Golob et al., 2021). Therefore, the properties of Douglas fir from North America are not comparable to those of Douglas fir from Europe (Blohm et al., 2014). The excellent reputation of Douglas-fir wood is due to the exceptional quality of wood from old-growth forests in north-western North America, which are characterised by slow growth rates (Gartner et al., 1999). However, the characteristics of wood from more dynamically growing second-growth forests in Europe may be different. European forest owners frequently bring smaller logs to market with a higher proportion of juvenile wood (Vikram et al., 2011). The higher proportion of juvenile wood could be problematic, as this wood has less favourable mechanical properties than mature wood (Pollet et al., 2017). On the other hand, faster growth might also result in different durability, as the growth reflects in the anatomy-related water performance and presence of biologically active secondary metabolites.

Wood is the biochemical product of living trees, which consists of basic building elements, i.e., cellulose, hemicelluloses and lignin, representing approx. 95% of the wood (Holmbom et al., 2008). Wood also contains smaller amounts of non-structural compounds, known also as extractives (Vek et al., 2020). Extractives include a large variety of compounds that can be removed from the wood tissue by relatively simple extraction methods. Although the amount of extractives in most of the wood species is relatively low, these non-structural components are reported to possess antifungal, antimicrobial and antioxidant properties (Lu et al., 2016; Belt et al., 2017; Valette et al., 2017). Phenolic extractives that increase the wood durability are biosynthesised *de novo* at the boundary between sapwood and heartwood (Magel et al., 1994; Dellus et al., 1997; Kebbi-Benkeder et al., 2017). Taxifolin and dihydrokaempferol are reported as such compounds for wood of Douglas fir (Dellus et al., 1997). It is known that the amounts of extractives in the wood of all trees, as well of Douglas fir vary (Adamopoulos et al., 2005; Vek et al., 2020). Specific woody tissues contain more extractives than others, e.g., the wood of dead branches embedded in a stem (wood of knots) is one of the richest sources of polyphenols in nature (Willför et al., 2004). The literature reviews on the chemical composition of extractives of Douglas fir shows that sapwood, heartwood, knotwood and bark have already been investigated (Willför et al., 2003; Miranda et al., 2021). Flavonoids, lignans and oligomeric polyphenols were the main phenolic compounds in wood extracts of Douglas fir (Lindberg et al., 2004; Brennan et al., 2021). Oleson and Schwartz (2016) have categorised proanthocyanidins, phlobaphenes, flavonoids, waxes, terpenoids, phytosterols and lignans as the most abundant non-carbohydrate extractives in the wood and bark of Douglas fir. This review also shows that heartwood contains higher amounts of proanthocyanidins and flavonoids than sapwood. On the other hand, Douglas-fir sapwood and heartwood were reported to contain comparable amounts of waxes, terpenoids and phytosterols (Dellus et al., 1997; Oleson and Schwartz, 2016). In comparison with heartwood, extraction of sapwood with a mixture of acetone and water yield higher amounts of flavonoid glycosides and procyanidins (Dellus et al., 1997). The two goals of our chemical analysis were first to complement the existing literature data and to investigate the chemical composition of phenolic extractives in Douglas-fir heartwood samples of different ages, and then to study the possible correlation between the results of chemical analysis and the results of fungal testing.

The purpose of the respective document is to present information about material resistance and moisture dynamics of Douglas fir from Slovenian forests. This information is of great importance for assessing the suitability of the selected wood species in outdoor applications.

## Materials and Methods

### Materials

In the area of Planina (45.818785 and 14.240381) and Celje (46.193462 and 15.269257), we cut three specimens per location (six trees in total) of Douglas fir (PsMe; *Pseudotsuga menziesii*). In both locations, coastal Douglas-fir (*P. menziesii* var. *menziesii*) provenances from Washington and Oregon were planted. Exact origin of seeds is not known. Recent genetic survey (Kraigher et al., 2021) revealed slightly lower genetic variation in these populations when compared to genetic variation in species’ native range but no significant differences between Planina and Celje. The trees in the respective locations were selected randomly. The trees were of comparable size, dimension and age on the location. They were representative ones of the assessed population. The breast diameter of the trees used ranged from 40 to 45 cm, and all trees were about 70 years old. Locations Planina and Celje were selected as they are among largest and most typical areas of high-quality Douglas-fir stands in the country. In addition, Douglas-fir stands in Planina have shown good resistance to ice storms, drought and bark beetle infestation in comparison with Norway spruce, and stands in Celje have shown excellent growth performance. The trees in Planina grew in a mixed Norway spruce-Douglas-fir plantation established on a beech-fir site on the limestone at an elevation of 560 m. In contrast, the trees near Celje grew in a mixed conifer forest composed of Norway spruce (60%), Douglas-fir (23%), silver fir (6%) and common beech (3%) on the limestone at an elevation of 620 m. There is no difference in silviculture measures as group shelterwood silvicultural system is used in both forests.

We used the lower part (4 m height) of the logs and sawed them on a horizontal sawing machine into prisms suitable for further analysis. The following part of the log was considered for the analysis: sapwood (SW), mature heartwood (MW) and juvenile heartwood (JW) of the Douglas-fir samples. Samples have full traceability. Each tree was analysed separately. However, as there were no considerable differences between the trees the data were merged on the location level. For comparison, we analysed the wood of Norway spruce (PiAb; *Picea abies*), Scots pine (PiSy; *Pinus sylvestris*) and European larch (LaDe; *Larix decidua*) from the same part of Slovenia as well. The wood originates from multiple trees as well.

### The Density and Modulus of Elasticity

The density of samples conditioned at standard laboratory climate [20°C; 65% RH (Relative Humidity)] was determined from the mass and dimensions of the samples measured with a digital calliper. The experiment was performed in 10 parallels. The modulus of elasticity (MoE) was determined according to the EN 310 (CEN, 1993) procedure with a static three-point bending test on a Zwick Z005 universal testing machine (Zwick-Roell). Samples (ten parallel samples for each tissue/tree) with dimensions 65_A_ mm × 25_T_ mm × 5_R_ mm were prepared and conditioned in a standard climate. Only MoE was determined, as the samples were consequently used to evaluate the terrestrial microcosms durability.

### Dynamic Vapour Sorption Analysis

The sorption isotherms of sapwood (SW), mature (HW) and juvenile heartwood (JW) of Douglas-fir samples were performed using a gravimetric dynamic sorption analyser (DVS Intrinsic, Surface Measurement Systems Ltd., London, United Kingdom). The milled samples were conditioned at 20 ± 0.2°C and 1 ± 1% RH for at least 24 h before the experiment. For analysis, a small amount (approximately 400 mg) of the sample was placed on the sample holder and suspended in a microbalance within a sealed, thermostatically controlled chamber in which a constant flow of dry compressed air was passed over the sample at a flow rate of 200 cm^3^/s and a temperature of 25 ± 0.1°C throughout the RH range. The DVS method was set to 20 steps of 5% between 0 and 95% RH for the sorption and desorption steps. Two total isothermal runs were performed to capture the sorption behaviour of the material fully; however, only one cycle is presented in the respective study. The instrument held the target RH constant until the rate of change in sample moisture content (dm/dt) was less than 0.002% per minute for 10 min. The run time, target RH, actual RH and sample weight was recorded every 20 s throughout the isothermal run. Adsorption and desorption isotherms were constructed by plotting the change in equilibrium moisture content (EMC) against relative humidity (RH).

### SEM Microscopy

Scanning electron microscopy (SEM) was performed to reveal detailed anatomical features of the three-dimensional structure of the wood. Samples were cut into 1 cm^3^ cube, ensuring that they were oriented in all three anatomical planes. The surfaces were planed using a sliding microtome equipped with a new disposable blade. Samples were dried above silica gel and in a vacuum and coated with gold (Q150R ES Coating System Quorum technologies, Laughton, United Kingdom) for 30 s at 20 mA intensity. SEM micrographs were then taken at high vacuum and low voltage (between 5 and 12.5 kV). A large field detector (LFD) and a concentric backscatter detector (CBS) were used in an FEI Quanta 250 SEM microscope (Hillsboro, Oregon, United States). Observations were made at a working distance between 8 and 11 mm.

### Analysis of Extractives

Wood extractives of Douglas fir were chemically investigated according to the methodology as already described (Singleton et al., 1965; Willför et al., 2004; Vek et al., 2019). Briefly, samples for chemical analysis were obtained from the same part of the wood as for the durability experiments. More sample discs were taken from each harvested tree of Douglas fir. One sample of sapwood and two heartwood samples, i.e., mature and juvenile heartwood, were pulled out from the stem discs using a band saw and chisel. The samples were then oven-dried (60°C, 24 h) and ground on a cutting mill Retsch SM2000 using a 1 mm sieve. Obtained wood fractions were then freeze-dried and extracted in Thermo Scientific ASE 350 system for speed extraction. Samples were extracted successively with cyclohexane and 95% acetone (v/v, aq) at 90°C and 100°C, respectively, and 10.34 MPa with two 5 min static cycles. Acetone has been demonstrated as a strong and ‘cheap’ solvent for extracting low-molecular-weight phenolic compounds from the wood of trees (Willför et al., 2006). The content of lipophilic and hydrophilic extractives was measured gravimetrically by oven drying 10 ml of an extract to constant mass. Total phenolic compounds in extracts were measured colourimetrically with Perkin Elmer Lambda UV–Vis spectrophotometer (Singleton et al., 1965; Vek et al., 2019). The Folin–Ciocalteu phenol reagent and sodium carbonate water solution were added to Douglas fir’s wood extracts. After incubation, the absorbance was measured at 765 nm. Solutions of gallic acid were used for calibration. The method for semi-quantitative analysis of total phenols in the extracts was linear for a selected concertation range (*R*^2^ > 0.99). A Thermo Scientific Accela HPLC system equipped with a PDA detector performed identifying and quantifying individual phenolic compounds in the wood extracts. Phenolic compounds were separated on the Thermo Accucore ODS column, and methanol and water with 0.1% formic acid were used as a mobile phase. Phenolic extractives were eluted out of the column, applying a linear gradient from 5 to 95% of methanol in 20 min. The eluted compounds were detected at 280 nm, and spectra were recorded from 200 to 400 nm. Identification of phenolic extractives was made utilising external analytical standards. The HPLC method for quantitative evaluation was linear (*R*^2^ > 0.99). The analytical standards, i.e., catechin, epicatechin, homovanillic acid, coumaric acid, taxifolin, secoisolariciresinol, pinoresinol and matairesinol, were of an HPLC purity, and they were all purchased from Sigma Aldrich. Each sample was injected into the column three times. All the results were expressed as milligrams of extractives per gram of dry material (mg/g, dw). The results were analysed with basic statistical analysis using Statgraphics software. ANOVA and Fisher’s least significant difference (LSD) procedure at a 95.0% confidence level were performed.

### Performance Against Blue Staining and Moulding

Samples of sapwood (SW) and mature heartwood (HW) of Douglas fir were exposed to blue-stain fungi according to EN 152 (CEN, 2012b) and fungi in condensing environments AWPA E24-12 (AWPA, 2015). Juvenile wood was not exposed due to a lack of material. The laboratory blue-stain test was conducted with the blue-stain fungi *Aureobasidium pullulans* (de Bary and Löwenthal) G. Arnaud strain ZIM L060 and *Sydowia polyspora* (Bref. and Tavel) E. Müll. strain ZIM L070. Both strains were obtained from the Collection of wood decay fungi from the Department of Wood Science and Technology (Biotechnical Faculty, Ljubljana, Slovenia; Raspor et al., 1995). Before inoculation, wood samples were sterilised in an autoclave with hot steam at 121°C and 150 kPa for 15 min. Later, the sterilised samples were immersed in a spore suspension and placed horizontally in a Kolle flask inoculated with 15 ml of spore suspension. The flasks were then stored in an incubation chamber at 25°C and 85% RH for 6 weeks. After this time, the samples were visually evaluated and ranked from 0 to 3 according to the ranking system prescribed in EN 152 (rank 0 = not blue-stained; 1 = small spots less than 2 mm; 2 = blue-stained up to one-third of the surface; and 3 = intensely blue-stained). Only the uppermost side was evaluated for the colour measurements.

The parallel samples were exposed to the condensing environment in the chamber. The main objective of this standard was to evaluate the resistance of the surface of wood samples to mould growth. The samples were exposed above water in the climatic chamber, which contained soil and a shelf for the test samples, with an inclined roof preventing condensation deposition on the samples. The growth of fungi on the wood samples was evaluated weekly. To allow comparison, the assessment followed the recommendations of the modified Johansson protocol (Johansson et al., 2012). Surface mould was evaluated using a 0 to 4 rating scale as follows:

0 = not blue-stained;

1 = weakly blue-stained: few spots of blue stain on the surface;

2 = slightly blue-stained: up to 1.5 mm wide and 4 mm long;

3 = moderately blue-stained: up to one-third of the surface; and.

4 = severely blue-stained.

### Determination of Factors Describing Inherent Durability (k_inh_)

Agar block tests with pure fungal cultures were used to evaluate inherent durability. A decay test was performed according to a modified CEN/TS 15083–1 procedure (CEN, 2005a). Samples (1.5 × 2.5 × 5.0 cm^3^) were conditioned in a standard laboratory climate (T = 25 ± 1°C; RH = 65 ± 2%) and steam-sterilised in an autoclave before incubation with decay fungi. A 350 ml experimental jars with aluminium lids and cotton wool with 50 ml potato dextrose agar (DIFCO, Fisher Scientific, Franklin Lakes, NJ, United States) were prepared and inoculated with the white-rot fungus *Trametes versicolor* (L.) Lloyd (ZIM L057) and two brown-rot fungi, *Gloeophyllum trabeum* (Pers.) Murrill (ZIM L018) and *Fibroporia vaillantii* (DC.) Parmasto (ZIM L037). The fungal isolates came from the fungal Collection of the Biotechnical Faculty of the University of Ljubljana and are available on request for research institutions (Raspor et al., 1995). One week after inoculation, two random samples per jar were positioned on high-density polyethylene (HDPE) plastic mesh to avoid direct contact between the samples and the medium. The assembled test jars were then incubated at 25°C and 85% relative humidity (RH) for 16 weeks as required by the standard. After incubation, samples were cleaned of adhering fungal mycelium, weighed to the nearest 0.0001 g, oven-dried at 103 ± 2°C and weighed again to the nearest 0.0001 g to determine mass loss due to wood-destroying basidiomycetes. Five replicate samples for each of the selected materials/wood species were used for this test.

In addition to agar block tests, resistance to soft-rot micro-fungi and bacteria was determined according to CEN/TS 15083–2 (CEN, 2005b). Ten replicate samples (5 mm × 10 mm × 100 mm) from each wood tissue and additional reference samples from Norway spruce were exposed to terrestrial microcosms. Samples were buried to 4/5 of their length in containers filled with non-sterile compost soil (origin: Ribnica, Slovenia) and stored at 27 ± 2°C and 70 ± 5% RH. The soil was used at 95% of its water holding capacity. The samples were exposed for 32 weeks. After exposure, samples were cleaned of adherent soil particles and mycelium, weighed to the nearest 0.0001 g, oven-dried at 103 ± 2°C for 24 h and weighed to the nearest 0.0001 g to calculate the mass loss. Afterwards, the samples’ modulus of elasticity (MoE) was determined using a three-point bending test according to EN 310 (CEN, 1993) before and after exposure to non-sterile soil. The MoE loss of the samples was calculated as a percentage of their initial values.

### Determination of Factors Describing Wettability (k_wa_)

A series of laboratory tests were performed to evaluate wettability. Tests were performed on five replicate samples (1.5 × 2.5 × 5.0 cm^3^) of each material. One set of samples was used for sorption tests and the other for various immersion tests. The average relative values of the different tests were combined to calculate the wettability factor as described by Meyer-Veltrup et al. (2017).

Short-term capillary water uptake was carried out at 20°C and 50 ± 5% RH, on a K100MK2 Tensiometer device (Krüss, Hamburg, Germany) according to a modified EN 1609 (CEN, 2013) standard after samples were conditioned to constant mass at 20°C and 65% RH. The axial surfaces of the samples were positioned so that they were in contact with the test liquid (distilled water), and their masses were then measured continuously every 2 s for up to 200 s. Other parameters used were as: speed before contact with the water 6 mm/min, the sensitivity of the contact 0.005 g and the immersion depth 1 mm. The water uptake was calculated in g/cm^2^ based on the final mass change of the immersed sample and the surface in contact with water.

Long-term water uptake was based on the leaching method ENV 1250–2 (CEN, 2004). Before testing, samples were oven-dried to constant mass at 60 ± 2°C and weighed to determine oven-dried mass. The dry wood blocks were placed in a jar and weighted to prevent floating. Then, 100 g of distilled water was added per sample. The mass of the samples was determined after 24 h, and the MC of five replicate samples was calculated. MC was determined gravimetrically as the ratio between the retained water and the oven-dried mass of the samples.

To determine the sorption properties of the samples, a water vapour uptake test was performed in a water-saturated atmosphere with a drying process over freshly activated silica gel. The samples were oven-dried at 60 ± 2°C to constant mass and weighed. The samples were stacked in a glass climate chamber with a fan over distilled water. The samples were positioned on mesh above the water with thin spacers (Meyer-Veltrup et al., 2017). After 24 h of exposure, they were weighed again, and the MC was calculated. The samples were then left in the same chamber for an additional 3 weeks until a constant mass was reached. In addition to wetting, outdoor performance is also influenced by drying. After the 3 weeks of conditioning, moist samples were positioned in a closed container over freshly activated silica gel for 24 h. The MC of the samples was calculated according to the procedure described by Meyer-Veltrup et al. (2017). Five replicate samples were used for this analysis.

### Factor Approach for Quantifying the Resistance Dose D_Rd_

A modelling approach based on Isaksson et al. (2014) and Meyer-Veltrup et al. (2017) was applied to predict the field performance of the examined materials. The model describes the climatic exposure and the resistance of the material. The acceptability of the chosen design and material is expressed as follows:


(1) Exposure≤Resistance

The exposure can be expressed as exposure dose (D_Ed_), determined by daily temperatures and MC. Material property is expressed as resistance dose (D_Rd_) in days (d), with optimal wood MC and wood temperature conditions for fungal decay (Isaksson et al., 2013):


(2) $$D_{\mathrm{Ed}}\leq D_{\mathrm{Rd}}$$

Where D_Ed_ is the exposure dose (d), and D_Rd_ is the resistance dose (d).

The exposure dose D_Ed_ depends on the annual dose at a given geographic location. Several factors describe the effect of driving rain, local climate, sheltering, distance from the ground and detailed design. Isaksson et al. (2014) describe the development of the corresponding exposure model in detail. The present study focused on the counterpart of the exposure dose, namely, the resistance, expressed as the resistance dose D_Rd,_ which is considered the product of the critical dose D_crit_ and two factors expressing the wettability of the wood (k_wa_) and its inherent durability (k_inh_). The approach is given by Equation (3) according to Isaksson et al. (2014; Table 1):


(3) $$D_{\mathrm{Rd}}=D_{\mathrm{crit}}\times k_{\mathrm{wa}}\times k_{\mathrm{inh}}$$

**Table 1:** Description of key terms addressed in respective article.

Term	Description
k_wa_	Factor describing the wetting ability of wood-based materials. The factor is expressed in relative values relative to the wetting ability of the spruce
k_inh_	Factor describing the inherent durability of wood-based materials. The factor is expressed in relative values relative to the inherent durability of the spruce
D_Rd_	Resistance dose reflects the material property and is expressed in days (d), with optimum wood MC and wood temperature conditions for fungal decay, before the first evidence of decay
Rel. D_Rd_	Relative resistance dose. Usually, spruce is used as the normalisation factor

Where D_crit_ is the critical dose corresponding to decay rating 1 (slight decay), according to EN 252 (CEN, 2012a), k_wa_ is a factor that considers the wettability of the tested materials [−], relative to the reference Norway spruce and k_inh_ is a factor that considers the inherent protective properties of the tested materials against decay [−], relative to the reference Norway spruce. Namely, the wettability and inherent durability of the Norway spruce were set to 1. Materials for which one of these values is better than that determined for Norway spruce have higher values overall but are limited to a value of 5.

Based on the results of the various moisture tests presented in this paper, the factor k_wa_ for wettability was calculated. The methodology for calculating k_wa_ followed that of the Meyer-Veltrup et al. (2017), except that the size of the samples differed. The original model prescribed samples (5 mm × 10 mm × 100 mm) that had a different shape than those used in the present study (15 mm × 25 mm × 50 mm). Since the methodology is based on relative values, the sample size has a minor influence on the results. The results of the durability tests were used to evaluate the inherent resistance factor k_inh_, and both factors were used to determine the resistance dose D_Rd_ of the wood materials investigated in this study.

## Results and Discussion

**Figure 1:**
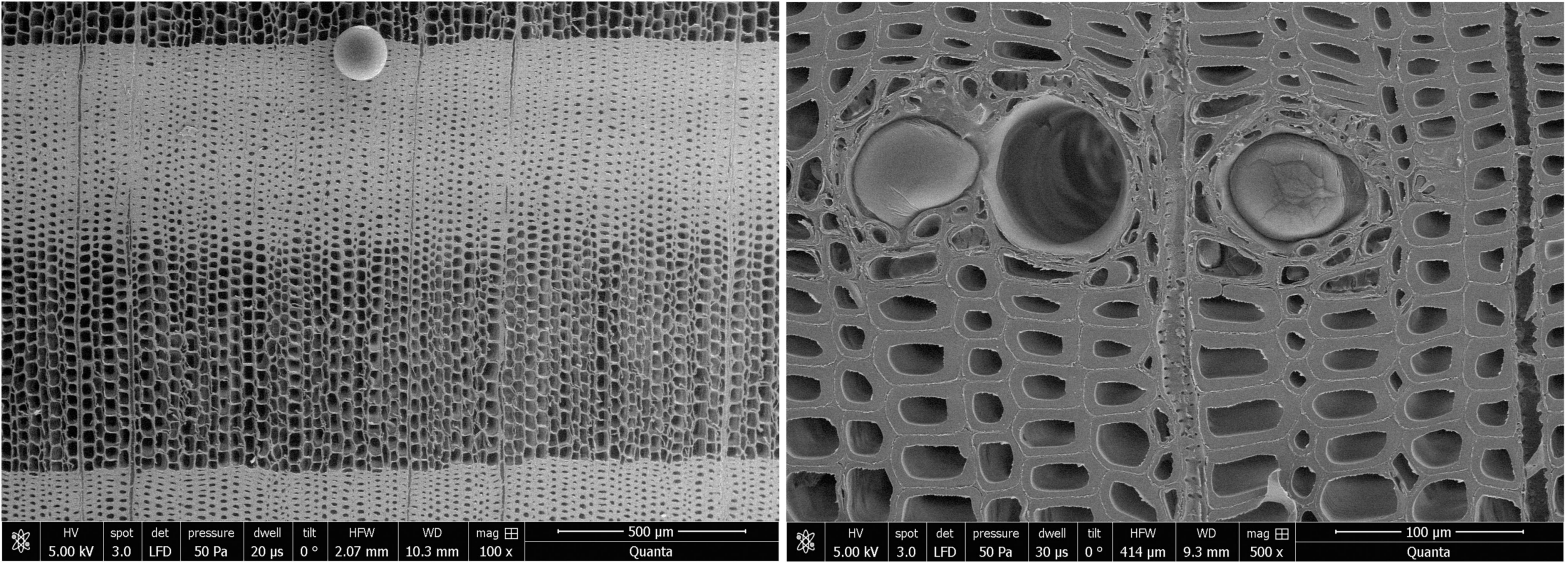
Annual ring of Douglas-fir mature heartwood (left) and resin canal in sapwood (right).

**Figure 2:**
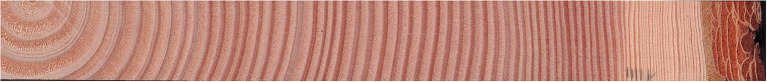
Cross-section of Douglas fir from Planina.

The anatomy of Douglas-fir wood is typical of conifers, with a sharp transition from early- to latewood. Resin canals with thick-walled epithelial cells are present (Wagenfuhr, 2007; Balanzategui et al., 2021; Figure 1). Annual ring widths varied from 1 cm in the juvenile wood to 1 mm in mature heartwood and in sapwood (Figure 2). Growth in the early years was fast compared to other softwoods growing in the region but comparable to growth rates of Douglas fir from other regions of Europe. In Belgium, for example the mean annual ring width varied from 3 mm to 8 mm (Pollet et al., 2017).

**Figure 3:**
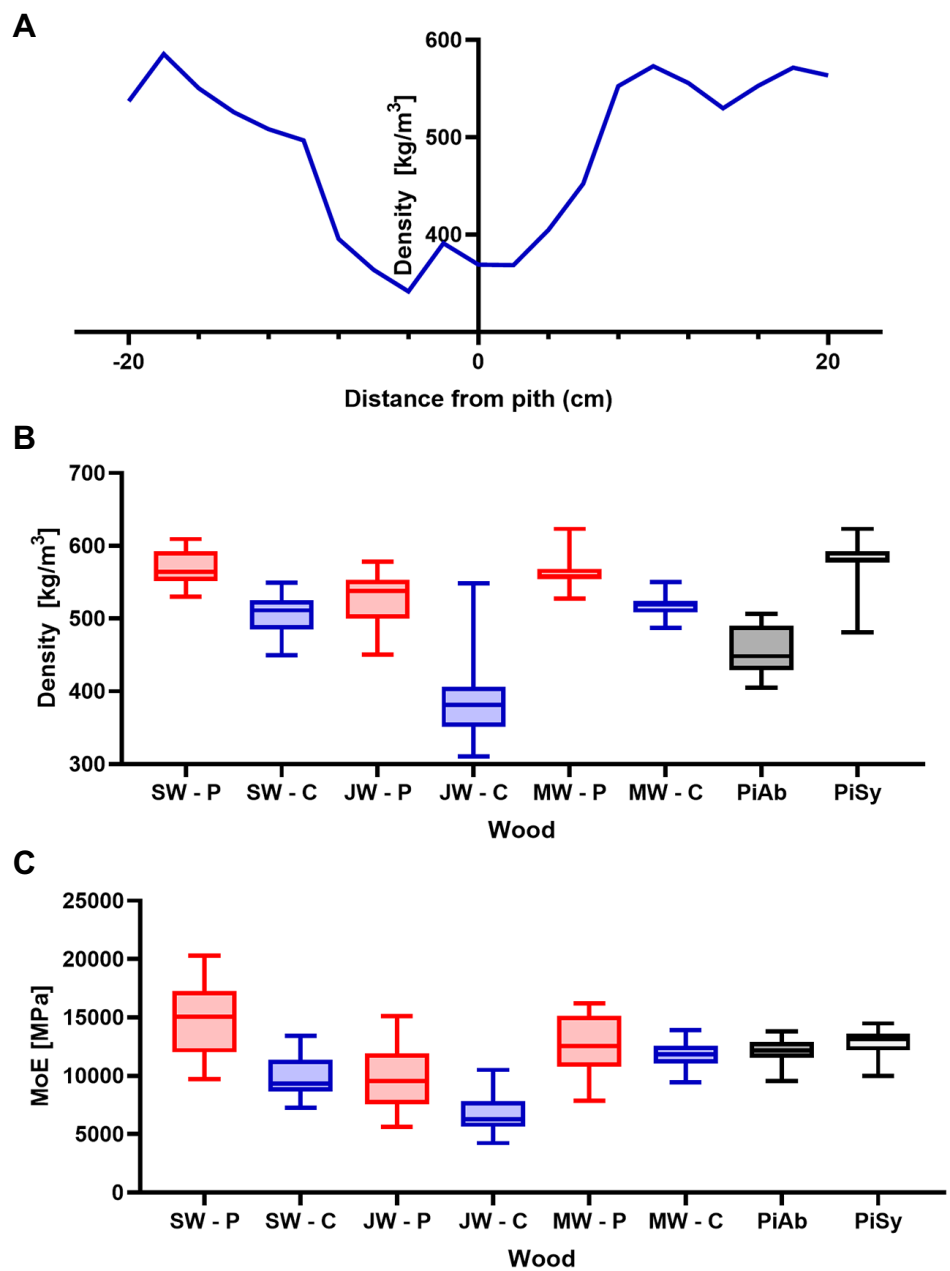
Relevant properties of the analysed wood species from various locations. **(A)** Density profile of Douglas fir from Planina. **(B)** Densities of Douglas fir from Planina (P) and Celje (C) and **(C)** Modulus of elasticities of Douglas fir from Planina (P) and Celje (C).

Significant differences in growth rates are also reflected in density. Densities of Douglas fir from Celje were lower than densities from Planina. The lowest density was measured in juvenile wood, which is consistent with the distribution of annual rings. The lowest density was determined by Celje’s Douglas-fir tree (342 kg/m^3^) with the broadest annual rings. The average density of Douglas-fir juvenile wood from Celje was 384 kg/m^3^, while the density of juvenile wood from Planina was slightly higher (527 kg/m^3^). The densities of juvenile wood are consistent with the literature data (Pollet et al., 2017). On the other hand, the density of mature wood was slightly higher, varying between 516 kg/m^3^ (Celje) and 568 kg/m^3^ (Planina). These values are also consistent with reference data for Douglas-fir wood (Wagenfuhr, 2007; Poletti et al., 2019). The density of Douglas fir is slightly higher than the average density of Norway spruce from Slovenia (457 kg/m^3^) and comparable to the density of Scots pine (578 kg/m^3^; Figure 3). The density and annual ring width are reflected in the MoE. Juvenile wood from Celje had the lowest MoE (6,766 MPa). In contrast, much higher values were obtained for mature wood and sapwood. The highest values were obtained for sapwood from Planina (14,724 MPa). The MoEs of mature heartwood from Planina (12,577 MPa) and Celje (11,882 MPa) are comparable to that measured on Norway spruce (12,024 MPa). The modulus of elasticity of mature Douglas-fir wood from Slovenia is comparable to the mechanical properties of Douglas-fir wood from Netherlands (Polman and Militz, 1996).

**Figure 4:**
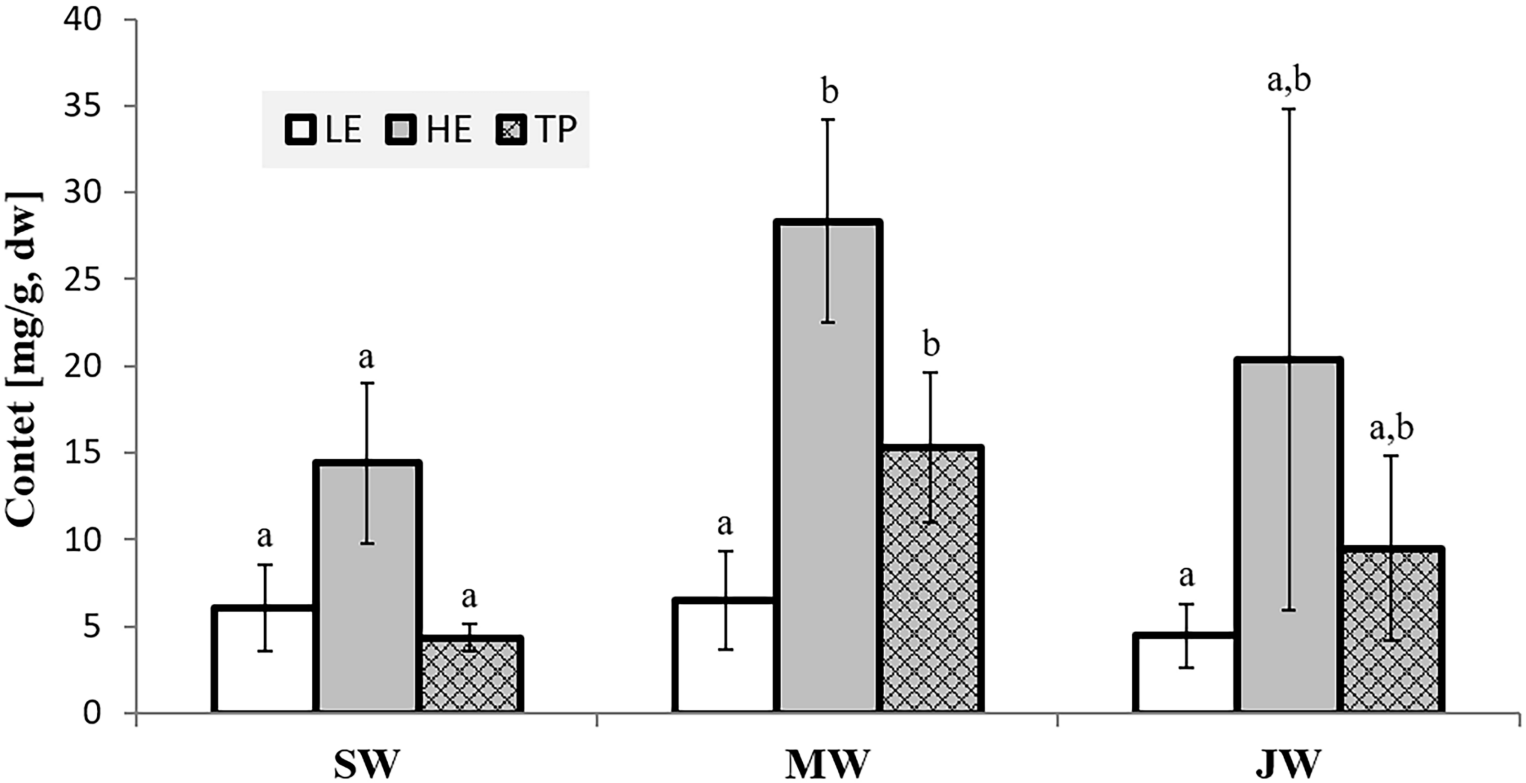
Content of in cyclohexane soluble compounds (LE, lipophilic extractives), in acetone soluble compounds (HE, hydrophilic extractives) and content of total phenolic compounds (TP) in the wood of Douglas fir (*Pseudotsuga menziesii*). Different letters on the top of the error bars indicate statistically significant differences at 95% confidence level. SW, sapwood; aHW, adult heartwood; jHW, juvenile heartwood.

**Figure 5:**
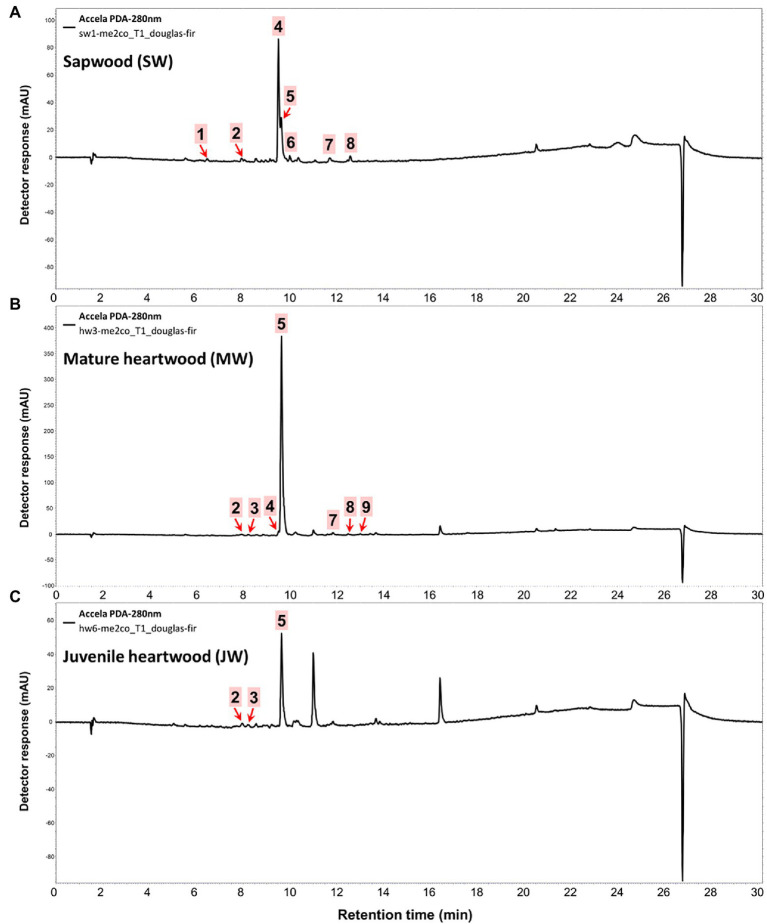
HPLC chromatograms of acetone extracts from **(A)** sapwood, **(B)** adult heartwood and **(C)** juvenile Douglas fir (*Pseudotsuga menziesii*). For peak assignment, see Table 2.

The amounts of extractives in the wood samples were measured to be 2.05% (SD = 0.42) for sapwood (SW), 3.49% (SD = 0.56) for mature heartwood (MW), and 2.48% (SD = 1.63) for juvenile heartwood (JW). Statistical analysis revealed that MW samples’ extraction gave significantly larger extractives than SW samples (LSD test). The content of measured lipophilic extractives was low, and the differences among the samples were not statistically different (ANOVA, *p* > 0.05; Figure 4). It was found that MW samples contain significantly larger amounts of hydrophilic extractives and total phenolic extractives than SW samples (LSD test; Figure 4). Even though the numbers were higher for the MW samples, significant differences in the content of hydrophilic extractives between MW and JW samples were not statistically confirmed (LSD test; Figure 4). The content of phenolic extractives in SW, MW and JW samples was measured to be 0.53% (SD = 0.09), 1.62% (SD = 0.43) and 1.03% (SD = 0.54). The differences in the contents of total phenols among SW, MW and JW samples were statistically significant (ANOVA, *p* < 0.01). These results correspond to the estimates for amounts of flavonoids present in sapwood and heartwood od Douglas fir, i.e., 0.5 and 2% of the oven-dried mass, respectively (Oleson and Schwartz, 2016). The HPLC analysis confirmed presence of catechin (*tr* = 6.5 min), epicatechin (*tr* = 8.0 min), homovanillic acid (*tr* = 8.2 min), coumaric acid (*tr* = 9.5 min), taxifolin (*tr* = 9.6 min), ferulic acid (*tr* = 10.0 min), secoisolariciresinol (*tr* = 11.7 min), pinoresinol (*tr* = 12.5 min) and matairesinol (*tr* = 13.0 min; Figure 5). These compounds have already been reported to be present in stem wood and knotwood of Douglas fir (Willför et al., 2003; Lindberg et al., 2004; Oleson and Schwartz, 2016). Isolariciresinol, lariciresinol and nortrachelogenin have also been reported to be present in smaller amounts in wood extracts of Douglas fir (Willför et al., 2003; Brennan et al., 2021).

The chemical composition of extractives presents in bark and knotwood of Douglas fir have already been comprehensively investigated (Miranda et al., 2021). However, there is hard to find exact information on individual phenolic compounds present separately in sapwood and heartwood (Dellus et al., 1997). Literature data on differences in the composition of extractives between mature and juvenile heartwood are completely missing. The measured quantities of the identified compounds are presented in Table 2. Taxifolin was found and confirmed as the most abundant compound present in the wood extracts of Douglas fir (Figure 5). The differences in the content of taxifolin among the sapwood and heartwood samples were statistically significant (ANOVA, *p* < 0.05; Table 3). The highest amounts of taxifolin gave the extraction of MW. The lowest amounts were measured in SW samples (LSD test) (Figure 5). The coumaric acid and especially ferulic acid were found to be characteristic for sapwood samples. These two simple phenolic acids were reported to be characteristic also for the resin of Douglas fir (Holmbom et al., 2008; Figure 5). Extraction of sapwood and heartwood samples gained somehow comparable amounts of lignans secoisolariciresinol and pinoresinol with no significant differences in the contents among the samples (ANOVA, *p* > 0.05, LSD test). Matairesinol was measured in the wood extracts in traces (Table 2). As well, catechin, epicatechin and homovanillic acid were present in the wood extracts of Douglas fir in traces (Table 3). Wood and bark extracts of Douglas fir are known to have antimicrobial properties (Lindberg et al., 2004; Välimaa et al., 2007). Acetone extractives of Douglas-fir knotwood were also demonstrated to possess a high antioxidative potency and radical scavenging capacity (Willför et al., 2003). In the frames of the study, taxifolin was found as a phenolic compound with high antioxidative potency and radical scavenging capacity (Willför et al., 2003). Taxifolin is also reported as a *de novo* synthesised compound of Douglas-fir heartwood that increases wood durability (Dellus et al., 1997). Higher resistance of the mature heartwood of Douglas fir to the fungal decay (Table 4) could be therefore explained by higher amounts of phenolic extractives and taxifolin in these tissues.

**Table 2:** Chemical composition of phenolic extractives in sapwood and heartwood of Douglas fir (*Pseudotsuga menziesii*).

Compound	Chemical structure	Peak no.	SW		aHW		jHW	
			*Avg*	*SD*	*Avg*	*SD*	*Avg*	*SD*
Catechin	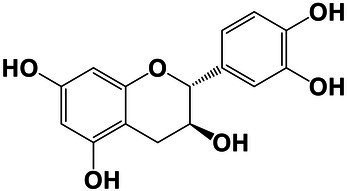	1	NQ < 0.2		NQ < 0.2		NQ < 0.2	
Epicatechin	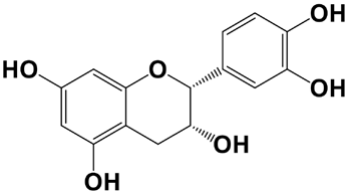	2	NQ < 0.2		NQ < 0.2		NQ < 0.2	
Homovanillic acid	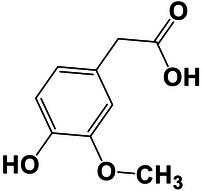	3	NQ < 0.2		NQ < 0.2		NQ < 0.2	
*p*-Coumaric acid	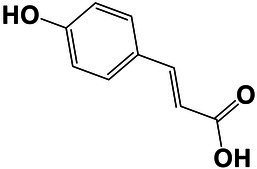	4	1.26	0.49^a^	0.78	0.31^a^		
Taxifolin	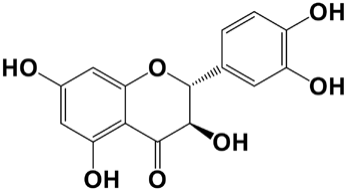	5	0.48	0.25^a^	6.45	5.19^b^	3.61	2.70^a,b^
Ferulic acid	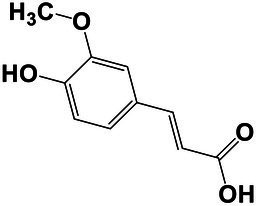	6	0.47	0.09				
Secoisolariciresinol	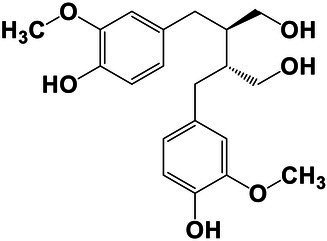	7	0.60	0.38^a^	0.43	0.34^a^	0.89	
Pinoresinol	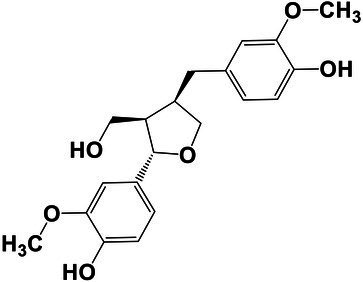	8	0.17	0.04^a^	0.27	0.13^a^	0.49	0.42^a^
Matairesinol	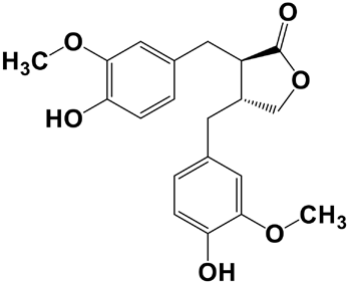	9	NQ < 0.2		NQ < 0.2			

Results are expressed by the mean value of measurements (Avg) and the standard deviation (SD). Different letters within the same row indicate statistically significant differences at a 95.0% confidence level. NQ < 0.2; identified in the extracts present in traces. The contents measured below 0.20 mg/g (dw); SW, sapwood; aHW, adult heartwood; jHW, juvenile heartwood.

**Table 3:** Factors that determine the service life of the wood.

Wood species	Tissue	Growth location	k_wa_	k_inh_	D_Rd_ (d)	D_Rd rel_
PiAb	Heartwood	Slovenija	1.0	1.0	325	1.0
PiSy	Sapwood	Slovenija	0.7	1.0	245	0.8
PsMe	Sapwood	Celje	1.0	1.3	436	1.3
	Sapwood	Planina	1.1	1.3	481	1.5
	Mature heartwood	Celje	2.2	2.9	2007	6.2
	Mature heartwood	Planina	1.8	2.6	1,500	4.6
	Juvenile heartwood	Celje	2.1	2.3	1,589	4.9
	Juvenile heartwood	Planina	2.0	2.6	1,652	5.1

The factors are the mean values of the individual factors calculated according to the methodology described in detail in Meyer-Veltrup et al. (2017) and Table 1.

**Table 4:** Moisture and decay indicators used to calculate factors that determine the service life of the wood.

Indicator	PiAb	PiSy	PsMe					
			Sapwood		Heartwood			
	AW	SW	SW	SW	MW	MW	JW	JW
	/	/	Celje	Planina	Celje	Planina	Celje	Planina
RH100 24 h (%)	16.8^a^	15.7^a^	15.8^a^	13.6^b^	13.7^b^	12.4^c^	17.2^a^	12.5^c^
RH100 (%)	28.7^a^	27.7^a^	27.1^a^	28.1^a^	25.2^a^	26.5^a^	27.2^a^	27.1^a^
Release (%)	8.6^a^	8.9^a^	7.3^a^	7.4^a^	6.4^b^	6.6^b^	8.3^a^	7.2^a^
STWU (g/cm^2^)	0.1234^b^	0.9528^a^	0.0981^b^	0.1185^b^	0.0210^c^	0.0428^c^	0.0189^c^	0.0331^c^
MC 1 h (%)	25.8^b^	52.7^a^	27.5^b^	18.1^c^	10.0^d^	8.5^d^	11.6^d^	8.0^d^
MC 24 h (%)	51.7^a^	67.4^a^	53.2a	40.5^b^	30.6^c^	25.9^c^	35.9^bc^	27.0^c^
								
Mass loss – GT (%)	35^a^	35^a^	27^b^	26^b^	14^c^	16^c^	22^bc^	16^c^
Mass loss – FV (%)	21^a^	23^a^	19^a^	20^a^	15^b^	14^b^	18^ab^	15^b^
Mass loss – TV (%)	20^a^	16^ab^	13^c^	12^c^	2^d^	4^d^	3^d^	2^d^
								
DC	5	5	4	4	4	4	4	4
Mass loss –TMC (%)	23^a^	26^a^	18^b^	17^b^	9^c^	10^c^	14^bc^	12^c^
MoE loss (%)	34.2^b^	48.4^a^	28.3^b^	32.6^b^	−2.9^a^	−2.7^a^	−1.6^a^	2.0^a^

RH100 24 h—water vapour uptake after 24 h, RH100—water vapour uptake after 3 weeks, release—water release after drying over silica gel, STWU—short-term water uptake determined with a tensiometer, MC 1 h—moisture content after 1 h of immersion and MC 24 h—moisture content after 24 h of immersion, mass loss after exposure to the respective fungi and terrestrial microcosm (TMC) and MoE loss after exposure to terrestrial microcosm. DC is a durability classification into durability classes according to CEN/TS 15083–1. Different letters indicate a statistically significant difference (*p* > 0.05) between different materials tested.

**Figure 6:**
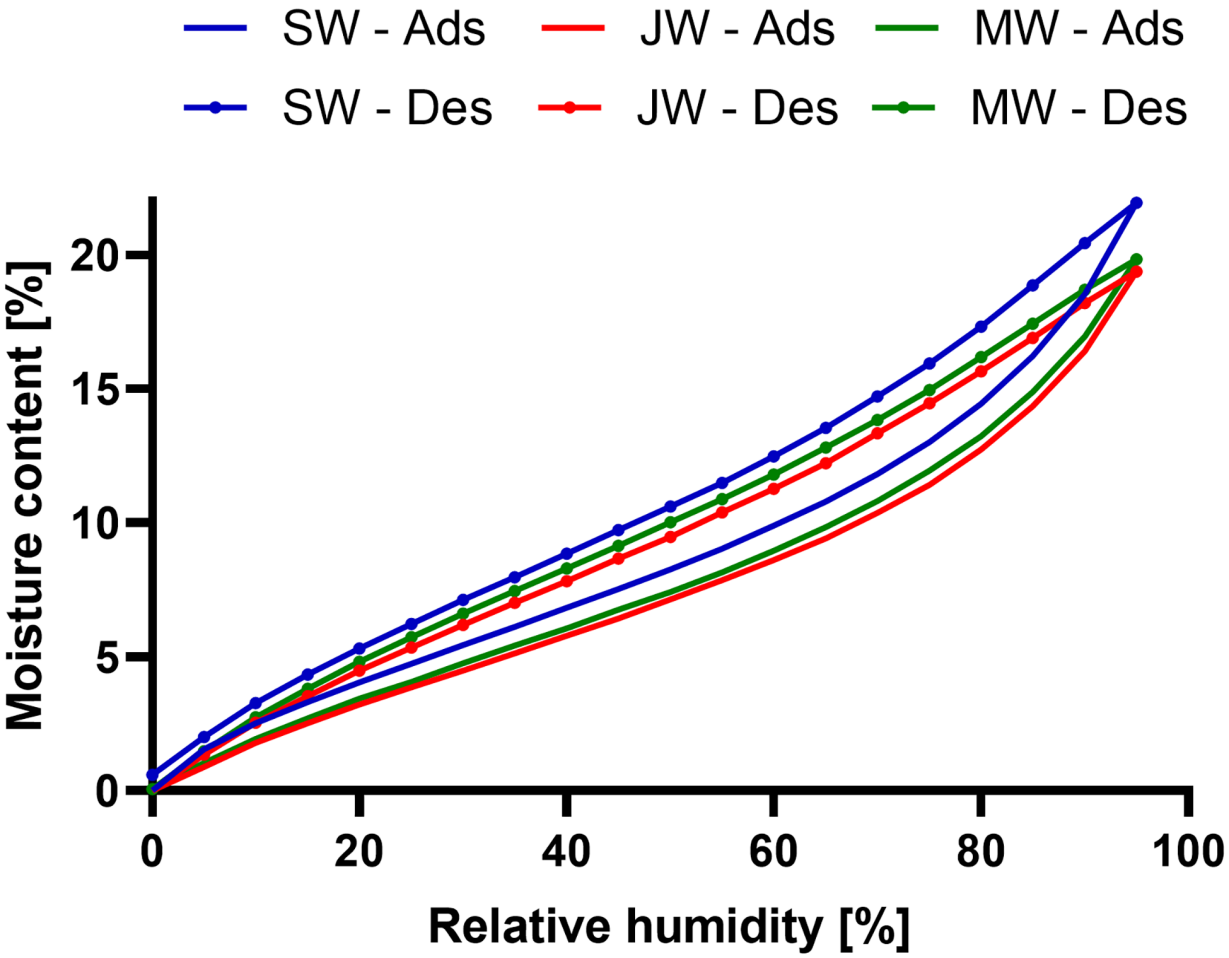
Relationship between relative humidity and wood moisture content. As determined with DVS for Douglas-fir sapwood (SW), Mature heartwood (MW) and juvenile heartwood (JW) of Douglas-fir samples in the second sorption (Sor) and desorption (Des) cycles.

The relative humidity is an important factor affecting the properties and performance of wood. When RH is high, wood absorbs water vapour from the air. The importance of air humidity increases when the air temperature is below or near the dew point. The relationship between wood MC and RH is shown in Figure 6. Wood MC increases with increasing RH. Since there was no significant difference between the Douglas-fir from Celje and Planina, only the results from Planina are shown. The surfaces of the tested wood samples are more polar than water molecules and therefore show increased water absorption at low relative humidities (0–10%). Once a single (mono-)layer of water is formed, the additional adsorption increasingly resembles the condensation of water. At high humidities, i.e., above 70%, adsorption is enhanced by tiny surface pores (mesopores with pore diameter 2 nm–50 nm). These attract water molecules on more than one side, i.e., capillary condensation. This leads to hysteresis in this humidity region caused by the reluctant release of adsorbed water (Mangel, 2000). There is no significant difference between juvenile and mature heartwood’s sorption and desorption curves. On the other hand, the moisture content of sapwood is higher. For example, the MC of heartwood at 95% RH ranged from 19.36 (juvenile heartwood) to 19.83 (mature heartwood). The moisture content of the sapwood was significantly higher (21.93%; Figure 6). This is consistent with the data in the literature (Jankowska et al., 2016). Chemical analysis shows that sapwood contains significantly fewer extractives than heartwood (Figure 4). Generally, wood species with higher extractives content have lower equilibrium moisture content (Hernández, 2007).

We determined the MC of wood exposed above the saturated water atmosphere for modelling purposes after 24 h of conditioning. The RH above saturated water ranges between 98 and 100%. This value reflects the ability of the wood to absorb water from the air. The reference Norway spruce wood absorbs 16.8% of the oven-dry mass in 24 h. On the other hand, a difference was found between the wood from Celje and Planina. On average, the MC of wood exposed above the water for 24 h from Planina is significantly lower than the MC of wood from Celje. For example, the MC of mature heartwood from Planina was 12.5%. In contrast, the MC of mature heartwood from Celje was comparable to the MC of spruce (17.2%; Table 4). These differences can be partially attributed to the different densities. Due to the higher porosity of wood from Celje, water vapour diffusion occurs more rapidly than in denser wood with lower porosity (Sjökvist et al., 2019).

In addition to water vapour, outdoor wood is frequently exposed to capillary water (Rice and Phillips, 2001; Žlahtič-Zupanc et al., 2018). Therefore, special attention was paid to assessing this part of water performance. Namely, two types of tests were performed, short-term water uptake, determined in axial planes and long-term water uptake, determined by long-term immersion of the samples, with water penetrating the samples in all directions. Special attention is paid to penetration in axial planes, as these surfaces are usually the weakest point for both water penetration and fungal attack (Žlahtič Zupanc et al., 2019). Short-term water uptake into sapwood (Celje 0.0981 g/cm^2^; Planina 0.1185 g/cm^2^) was comparable to that determined on Norway spruce wood samples (0.1234 g/cm^2^). On the other hand, the water uptake to Douglas-fir heartwood was much lower. Namely, water uptake of 0.0210 g/cm^2^ was determined for mature heartwood from Celje (Table 4). The main reason for the low uptake can be attributed to aspirated pits and the presence of extractives (Bolton and Petty, 1977). The same phenomenon can be observed in other conifers as well (Reinprecht, 2016). A similar effect was observed in samples immersed in water for 1 h. Water uptake in the Douglas-fir sapwood samples is in the same range as in spruce wood, while lower values were obtained in heartwood samples. The results of short- and long-term capillary water take also showed that water uptake was lower on samples from Planina than on samples from Celje (Table 4). The lower water uptake can be ascribed to the Planina samples’ higher density and lower porosity (Figure 3).

One of the main objectives of the manuscript was to determine the durability of the Douglas fir from Slovenia to wood-decaying fungi. The wood decay fungi used caused considerable mass loss of 35% (*Gloeophylum trabeum*) and 20% (*Trametes versicolor*) in the reference Norway spruce samples (Table 4). This indicates that the fungi were vital and that the wood was susceptible to fungal decay. Less decay of the Norway spruce samples exposed to the white-rot fungus *T. versicolor* is expected, as conifers are less susceptible to white-rot than brown-rot decay (Schmidt, 2006). The mass loss of the Douglas-fir sapwood after 16 weeks of exposure was slightly lower than the mass loss of the reference Norway spruce. Spruce wood exposed to *G. trabeum* lost 35%, while Douglas-fir sapwood samples lost between 27% (Celje) and 26% (Planina). A lower mass loss was observed in heartwood samples. Mature wood samples exposed to *G. trabeum* lost between 14% (Celje) and 16% (Planina). The *T. versicolor showed* a lower ability to degrade mature Douglas-fir heartwood, while the decay activity of *Fibroporia vaillantii* on heartwood was comparable to that of *G. trabeum* (Table 4). Better durability of the Douglas-fir wood can be ascribed to the higher concentration of biologically active extractives (Table 2).

Mass loss is a basis for the classification of durability according to the standard CEN/TS 15083–1 (CEN, 2005a). Based on the criteria of the respective standard, both sapwood and heartwood can be classified in durability class (DC) 4. Durability is on the borderline between DC 3 and DC 4, which is consistent with the standard EN 350 (CEN, 2016). Besides basidiomycetes, wood is frequently exposed to decay caused by terrestrial microorganisms, soft-rot fungi, and bacteria (Blanchette, 2000). Therefore, the parallel specimens were placed in the compost soil. The sapwood samples lost approximately 18% of the mass and 28% MoE. On the other hand, heartwood samples showed mass loss ranging from 9% (mature heartwood, Celje) to 14% (juvenile heartwood, Celje). However, the exposure did not result in significant losses of MoE. One of the possible explanations for this could be that the microorganisms eroded and abraded the surface, resulting in mass loss, but not loss of MoE.

Besides the decay fungi, the wood is also exposed to blue stain and sapstain fungi. All samples were completely covered with blue stains after laboratory exposure and rated with grade 3 according to EN 152 standard. On the other hand, exposure in the condensation chamber showed more significant differences (Table 5). The sapwood of Scots pine was covered entirely with sap stain after 5 weeks of exposure. On the other hand, fungal degradation progressed most slowly in Norway spruce wood. Spruce wood samples were rated 1 after 9 weeks of exposure. The disfigurement of Douglas-fir wood proceeded more rapidly. The sapwood reached an average value of 3.2 and the heartwood 2.2. The difference between the sites was not noticeable.

**Table 5:** Sapstain development in the condensing chamber in the 2 months between 19 May 2020 and 21 July 2020.

Wood species/date	PsMe				PiAb	PiSy
	Sapwood		Heartwood		Sapwood	
	Planina	Celje	Planina	Celje	Slovenia	Slovenia
26 May 2020	0.2	0	0	0	0	0
2 June 2020	1.6	2.2	0.4	0.6	0	1.2
9 June 2020	2	2.4	1	0.8	0.2	1.8
16 June 2020	2.2	2.6	1.2	0.8	0.4	3.2
23 June 2020	2.2	2.6	1.2	0.8	0.4	4
1 July 2020	2.6	3.2	1.4	1.2	0.6	4
7 July 2020	2.8	3.2	1.4	1.8	0.6	4
14 July 2020	2.8	3.2	2.2	2.2	1	4
21 July 2020	3.2	3.2	2.2	2.2	1	4

Factors describing wettability and inherent durability were calculated based on experimental analyses. The wettability of wood materials is a factor that indicates how the wood will behave during rain events. Wood that wets less and dries faster is less susceptible to fungal decay than wood absorbs more water during rain events and dries slowly. Sorption properties, permeability, possible tyloses formation, pit aspiration and other anatomically determined properties contribute to water penetration (Humar et al., 2020). As shown in Table 3, the moisture performance of Douglas-fir sapwood is comparable to that of Norway spruce. On the other hand, the k_wa_ factor of Douglas-fir heartwood is higher and is about 2 for both juvenile and mature heartwood, comparable to the moisture performance of European larch (Humar et al., 2019). It is known that the moisture resistance of heartwood is better than that of sapwood. In conifers, this is primarily due to pits aspiration and the process associated with heartwood formation (Bolton and Petty, 1977).

In addition to moisture performance, the k_inh_ factor, which characterises resistance to microbiological degradation, also plays a significant role in the performance of wood in outdoor environments. This durability primarily reflects the presence of biologically active extractives and biocides. Like the k_wa_ factor, the k_inh_ factor is also limited to 5. The durability of Douglas-fir mature heartwood varies between 2.9 (Celje) and 2.6 (Planina), which is slightly better than the value determined for European larch wood (1.8; Humar et al., 2019).

Based on the procedure proposed by Meyer-Veltrup et al. (2017), the material resistance dose (D_Rd_) was calculated assuming a critical dose independent of wood species (D_crit_ = 325 d). This number corresponds to the number of days with optimal conditions for fungal decay before the first signs of deterioration appear. The resistance dose of Douglas-fir sapwood (436 days—Celje; 481 days—Planina) was higher than that of the reference Norway spruce. A much higher resistance dose was determined in the heartwood. The corresponding value ranged from 2007 days (mature heartwood from Celje) to 1,500 days (mature heartwood from Planina; Table 3). The difference between juvenile and mature heartwood was not significant. These values indicate that Douglas-fir heartwood will perform much better outdoors than the reference Norway spruce wood. The analysis indicated that the main reasons for the observed difference are the better water performance and inherent durability of Douglas fir. In contrast to the traditional durability classification, the dosimeter-based resistance model applied here showed the excellent performance of Douglas-fir heartwood, indicating that this material can be used for outdoor applications.

## Conclusion

The average density of Douglas fir from two Slovenian sites was 489 kg/m^3^ (sapwood), 507 kg/m^3^ (mature heartwood) and 380 kg/m^3^ (juvenile heartwood). The low density of the juvenile heartwood can be attributed to the fast growth in the first decade.

The chemical analysis revealed the highest amounts of hydrophilic extractives and phenolic compounds in younger heartwood parts, i.e., mature heartwood. These tissues also contain the highest amounts of taxifolin. Higher resistance of mature heartwood against wood-decaying fungi can be therefore explained with the higher content of phenolic extractives and taxifolin.

The durability of Douglas-fir wood heartwood was like the durability of European larch and can be classified in the group of less durable wood species (DC 4). On the other hand, Douglas-fir heartwood exhibits good water exclusion efficacy, which contributes to a high resistance dose that can be attributed to pit aspiration. Based on durability and moisture performance tests, factors affecting inherent durability and wetting ability were calculated for Douglas fir. Douglas-fir sapwood exhibited a higher resistance dose (D_Rd_ = 459) than Norway spruce (D_Rd_ = 325). An even higher resistance dose was noted in Douglas-fir heartwood. There was a minor difference between juvenile (D_Rd_ = 1,621) and mature (D_Rd_ = 1754) heartwood. This indicates that Douglas fir has potential for outdoor use, where it can at least partially replace European larch and Norway spruce. As the population of Douglas fir in Slovenia is one of the southernmost populations in Europe, it can be expected that the performance of the Douglas fir from locations north of Slovenia will become more and more comparable to the values presented in the respective article because of the climate changes. Data presented herein can be used as an input factor for the prognosis of the future properties of Douglas fir from northern and central Europe.

## Data Availability Statement

The raw data supporting the conclusions of this article will be made available by the authors, without undue reservation.

## Author Contributions

MHu and VV designed the study and wrote the manuscript. BL, DK, VV, EK, and MHo carried out the experiments. RB found suitable trees and organised the harvesting of the trees. MHu and RB were responsible for project management. All authors contributed critically to the drafts and gave final approval for publication.

## Funding

The study was supported by Slovenian Research Agency (ARRS) within research program P4-0015 (Wood and lignocellulosic composites) and the infrastructural centre (IC LES PST 0481–09). Part of the published research was also supported by the Ministry of Agriculture, Forestry and Food in the frame of the project V4-1818.

## Conflict of Interest

The authors declare that the research was conducted in the absence of any commercial or financial relationships that could be construed as a potential conflict of interest.

## Publisher’s Note

All claims expressed in this article are solely those of the authors and do not necessarily represent those of their affiliated organizations, or those of the publisher, the editors and the reviewers. Any product that may be evaluated in this article, or claim that may be made by its manufacturer, is not guaranteed or endorsed by the publisher.
